# The Hospitals' Huge Contribution to the Cost of the War

**Published:** 1919-11-08

**Authors:** 


### KING EDWARD'S HOSPITAL FUND FOR LONDON.

### The Hospitals' Huge Contribution to the Cost of the War.

ONE of the outstanding features of the statistical report of the King's Fund for last year is the statement in the prefatory note showing the increase of ordinary expenditure of the hospitals of London between 1913 and 1918. The gross ordinary expenditure of 1913 was £1,142,938, that of 1918 was 1,946,155, an increase of over 70 per cent., but the editor of the report points out that as a sum of £334,747 was in 1918 paid by the naval and military authorities for Service patients treated in the wards, the ordinary expenditure falling upon the normal sources of hospital income was only £1,611,408 and the increase of ordinary expenditure was therefore 40.98 per cent. above that of 1913.

There is little doubt, in view of the heavy waiting-lists, that the beds of the hospitals, whether used for sailor and soldier patients or not, would have been kept occupied during the years of the war; there might even, judging from former experience, have been a small increase in their number. Therefore, to obtain a figure which has any practical value we must ascertain what the normal number of beds cost to maintain in 1918. The average number of beds occupied increased from 9,766 in 1913 to 11,566 in 1918. These 1,800 extra occupied beds may be counted as having been provided for Service patients (who altogether accounted for 3,685 beds.) The balance were beds that would have been occupied in any case. The true basis of calculation would be that 1,800 parts of the gross expenditure when divided by 11,566 was expenditure that would not have been incurred if there had been no sailor and soldier patients, and the balance (9,766 parts) is the expenditure of the hospitals running on ordinary lines, or the figure from which we may estimate the increased cost of hospitals at the end of the years of war.

### A CURIOUS STATISTICAL DISCOVERY.

Proceeding on these lines we find that the gross cost of running the previous establishment of the hospitals in the light of the 1918 scale of expenses was £1,643,277 which figure has some resemblance to the figure provided by Mr. Maynard's method —namely, £1,611,408. This near approximation leads to a curious discovery which probably was not in the mind of Mr. Maynard when he wrote his prefatory note and certainly not in that of the present writer when he commenced to examine the figures.

There was a strong suspicion in the minds of hospital administrators during the war that the capitation grant paid for Service patients was too small. This led to a request for a revision of the 5s. per day grant which gained an increased grant of 5s. 9d. per day, which was applied to the last year of the use of beds. Our review of the figures now reveals for the first time an indication of the extent of what the voluntary hospitals did for the Government without payment.

| | £ |
|---|---|
| 1. The gross ordinary expenditure of the hospitals in 1918 was ... ... ... ... | 1,946,155 |
| 2. Taking it that the extra beds that had been put up were on account of naval and military patients, and deducting their average cost, the gross ordinary expenditure of the hospitals on a pre-war scale of accommodation was ... ... ... ... ... ... | 1,643,277 |
| 3. The difference between the two sums is the cost of the extra 1,800 beds ... ... | 302,878 |
| 4. But the average number of beds occupied by Service patients in 1918 was 3,685. | |
| 5. The Government paid the hospitals of London during 1918 ... ... ... | 334,747 |
| 6. This sum defrayed the cost of the 1,800 emergency beds and left a balance of... ... to pay for the 1,885 beds taken from the normal establishment. | 31,869 |
| 7. These 1,885 beds, calculating on the same average of all the beds, cost ... ... | 317,179 |
| 8. Therefore the hospitals of London made a present to the Government of ... ... in a single year. | 285,310 |

In examining these figures it must be remembered that the definition of ordinary expenditure in the statistical report is expenditure on maintenance, administration and rent, rates and taxes, but not interest on loans, contributions in aid of convalescent homes or country branches, or capital expenditure.

### GIGANTIC GIFT TO THE STATE.

In fixing the grant the theory of the Government authorities was that the expense they had to meet was that which the hospitals were out of pocket on maintenance because of the reception of the patients, the expenditure on standing charges having to be met in any case. This was not considered by hospital authorities to be a satisfactory way of dealing with the matter, but, in the light of the above definition of ordinary expenditure, it will be seen that some standing and other charges came within the stated cost and some did not. Other points are that there was quite unusual wear and tear of furniture and general equipment, and that the Service patients were, by the desire of the authorities, given a more liberal diet than the civilian patients.

Our figures show that in respect of the 109 hospitals alone (those of London) a gift was made to the Government of the important sum of £285,310 in a single year. What then was the contribution made by the voluntary hospitals of the United Kingdom to the cost of the war during the four years of its duration?

We hope to deal in a second article with the routine matters covered by the report.

